# The Metabolic Effects of Ketones

**DOI:** 10.3390/ijms22158292

**Published:** 2021-08-02

**Authors:** Benjamin T. Bikman, Kelsey H. Fisher-Wellman

**Affiliations:** 1 Department of Physiology and Developmental Biology, Brigham Young University, Provo, UT 84602, USA; 2 Department of Physiology, East Carolina University, Greenville, NC 27858, USA; FISHERWELLMANK17@ECU.EDU

The phrase “once trash, now a treasure” is an apt description of the evolving view of ketones in biomedical research. Produced in states of low insulin and high fatty acid oxidation, ketones are either a dreaded sign of poorly controlled diabetes, or alternatively a sought-after signal for those seeking improvements in certain conditions. Regardless of the circumstances, ketones are energetic molecules that, in addition to providing calories to almost all cells, act as robust signaling molecules in clinically relevant processes, including inflammation [1] and oxidative stress [2]. 

Ketones, including acetoacetate and β-hydroxybutyrate (β-HB), stand at the axis of biochemistry and physiology, altering fundamental biochemical processes and, perhaps as a result, altering the physiology of a cell to often improve function. Given the explosive growth in ketone-related research, new findings from this humble family of nutrients is likely to only grow. 

To facilitate this interest, we sought to elucidate novel facets of ketone biochemistry and their relevance in cell biology across two separate Special Issues entitled “The Effects of Ketones on Metabolic Function” in *International Journal of Molecular Sciences*. Collectively, this yielded eight manuscripts, including five original research articles, two communication letters, and one review. 

We were very pleased and honored to publish a communication from Dr. Richard Veech [3]. To our knowledge, this was the final publication from the Veech lab prior to Dr. Veech’s passing. This is relevant insofar as Dr. Veech was a pioneer in the realm of ketone research, with his work resulting in hundreds of manuscripts, most of which contributed significantly to our understanding of the biochemistry of ketone metabolism, particularly in the brain. Indeed, this was the topic of his lab’s publication, wherein he and his group detail the effects and potential application of novel exogenous ketones in correcting behavioral and cognitive anomalies. 

Licha et al. [4] explored the novel use of ketogenic diets in mitigating the growth of cancers in a xenograft breast cancer rodent model. With this model, they found “clear evidence” that a ketogenic diet stunted tumor growth, suggesting that a ketogenic diet alters the bioenergetics of cancer cells, resulting in diminished growth. 

Acetoacetate is considered the “mother ketone”, the main ketone from which the alternate ketones are born (i.e., β-HB and acetone). Citing previous work that has used lithium acetoacetate as an anti-tumor molecule, Vidali et al. [5] challenged the role of the acetoacetate, finding instead that lithium acts alone. 

A common refrain among ketone-focused scientists is “ketones are muscle sparing”. Traditionally, this has referred to the phenomenon whereby ketones act as a fuel for the brain, effectively replacing what would otherwise be a high glucose demand coming from muscle amino acids (via gluconeogenesis). However, Parker et al. [6] find that ketones elicit direct and favorable changes at the muscle cell, including reducing mitochondria-derived oxidative stress. 

G protein-coupled receptors (GPR) mediate myriad biochemical processes, and some, namely GPR109a, even respond to ketones. Given the numerous benefits elicited in response to fasting, Geisler et al. [7] sought to determine the degree to which ketone binding GPR109a mediates some of these benefits. By using GPR109a wild-type or knockout mice, they revealed that GPR109a is not a necessary mediator in these events. 

Following a similar line from Dr. Veech’s lab, Suissa et al. [8] explored the effect of a ketone ester in altering brain mitochondrial bioenergetics in mice. Among their several results, they found that exogenous ketones (via the ketone ester) increased brain citrate cycle intermediates, suggesting greater available energy. Further, they showed that higher ketones resulted in diminished brain glucose use, hinting at a competitive use and even perhaps a fuel preference (i.e., ketones over glucose). In the end, they provide evermore evidence that the ketone ester should be considered in the therapeutic approach to neurological disorders. 

The global rise in obesity and related disorders has health professionals on the hunt for effective strategies for mitigation. Walton et al. [9] provided compelling evidence of a direct metabolic benefit from ketones. By expansively testing cultured adipocytes, rodent adipose tissue, and human adipose biopsies, they found that ketones directly increase adipocyte mitochondrial uncoupling, resulting in increased metabolic rate within fat cells. These results could partly explain why ketogenic diets increase metabolic rate in humans [10,11]. 

Jensen et al. [12] provided a thorough review of the state of knowledge regarding ketones and neurodegenerative diseases. This review highlighted the high use of ketones as a fuel for the brain, and further elucidated the growing number of clinical strategies that seek to leverage these findings. 

In the end, the editors are delighted to have worked with *International Journal of Molecular Sciences* to bring this Special Issue to publication. The editors thank the contributors to this Special Issue—their commitment and enthusiasm for this topic continue to drive the field. Furthermore, the responsive and incredibly helpful staff at Multidisciplinary Digital Publishing Institute (MDPI) worked tirelessly to ensure this Special Issue was a successful venture. 
